# Skull Abnormalities in Cadavers in the Gross Anatomy Lab

**DOI:** 10.1155/2020/7837213

**Published:** 2020-02-19

**Authors:** Jessica De Rose, Brian Laing, Maha Ahmad

**Affiliations:** ^1^University of Detroit Mercy School of Dentistry, USA; ^2^Division of Integrated Biomedical and Diagnostic Sciences, University of Detroit Mercy School of Dentistry, USA

## Abstract

**Background:**

The skull encompasses and houses one of the most important organs in the body—the brain—and like all tissues in the body, it is comprised of living cells that are constantly remodeling as this maintains the strength and homeostasis of the bone. In the present study, abnormal bone growth patterns were observed and the possible causes of said findings were investigated in multiple cadaver skulls dissected during head and neck anatomy courses at Detroit Mercy Dental over the past year. There are many factors, both intrinsic and extrinsic, with differences in stimulation to the skull resulting in skull abnormalities. *Materials and Methods*. For this study, skull abnormalities were examined from 65 formalin-embalmed cadaver heads, obtained from the Gross Anatomy Laboratory at the University of Detroit Mercy School of Dentistry between the years 2016 and 2019. We have recorded the age, sex, and previous chief medical issues of all lab specimens used in the study. Skulls were later evaluated for possible indications of bone disease such as hypertosis frontalis interna (HFI) or Paget's disease.

**Results:**

Among the sixty-five specimens provided to the Detroit Mercy Dental cadaver lab, 19 specimens (29%) were found to present with irregular, undulating, thickening of the frontal bone internal surface. The findings located on the skulls closely resembled the gross anatomic appearance of HFI or Paget's disease; however, a conclusive diagnosis of these skull abnormalities cannot be made without a pathologist biopsy and radiological examination. Twelve of the nineteen specimens that displayed possible bone disease, approximating 63% prevalence, were females; their ages ranged from 68 to 95 years old. Thus, seven of the nineteen specimens exhibiting features of skull abnormalities, approximating 36% prevalence, were males with ages ranging from 70 to 103 years old. In addition, five of these nineteen specimens collected (26% prevalence) had been diagnosed with neurological disorders, including Alzheimer's, dementia, depression, and Parkinson's disease. In the current study, the proportion of specimens exhibiting skull abnormalities was higher compared to the overall prevalence observed in previous studies.

**Conclusion:**

Possible causes of observed anatomical abnormalities in the skull of cadavers of a gross anatomy laboratory were investigated, and it was determined that hypertosis frontalis interna (HFI) may contribute to such abnormalities. This is a condition that affects bone growth in the frontal skull. Our numbers of skull abnormalities were higher than previous studies and might be due to the fact that HFI was predominately present as an incidental finding during imaging of postmenopausal females or observed postmortem in cadavers. In addition, Paget's disease or hormonal imbalances could also result in similar features, and thus cannot be ruled out as a plausible cause. Paget's disease causes the bone to deposit at a faster rate than normal, which will result in thick and brittle bone. Studies that will involve further examination of new cadavers for the presence of HFI is needed, either using biopsy specimens and/or radiological examination to explore possible causes for the abnormal bone growth in the frontal bone.

## 1. Introduction

Mechanical stimulation influences the process of remodeling, and it has been documented that there is a decrease in cortical bone and an increase in trabecular porosity as adults age [1]. In addition, evidence suggests that with age the greatest loss in gray matter density occurs in the frontal and parietal regions of the brain and may correlate with thickness in the frontal and parietal bones as an attempt to balance the intracranial pressure [1]. Thus, a combination of both age and mechanical stimulation can influence the process of bone stimulation and structural changes seen in these populations.

In addition to external factors, there are internal factors that may influence the health and homeostasis of bone remodeling. Studies have also suggested a link between Alzheimer's disease and bone density. In a study performed by Loskutova et al. [2], it has been suggested that low bone mineral density can be correlated with Alzheimer's disease when compared to a group of healthy females of the same age. Many factors can be taken into account to explain the association, but some evidence suggests that the hypothalamus, a structure affected in Alzheimer's disease, regulates bone health and may play a larger role in the destruction of the bone density of the skull in said patients [2].

Furthermore, experimental data suggests that in an aging population there is a decline in one's motor adaptability, performance, and learning capabilities [3]. Thus, studies have been conducted to determine if an aging human brain has the same abilities to change its structural neuroplasticity in response to learning and/or practicing just as a 20-year-old does [3]. Given that any changes in the brain structure will consequentially result in changes in the structure of the skull to maintain intracranial pressure, the skull in postmortem cadavers can be interrupted to understand what factors may have caused such changes. The hippocampus, a structure involved in lifelong neurogenesis, has showed plasticity similar to the nucleus accumbens [3]. Physical activity and an enriched environment have the ability to improve neurogenesis in an older population and maintain the new neurons produced. Thus, differences seen in cadaver skulls in said areas may be due to how active the person was prior to death.

Research has also suggested a strong correlation between the thickening of the skull, particularly the frontal lobe, and the appearance of irregular undulations with hypertosis frontalis interna (HFI). Structural abnormalities can occur in the frontal lobe and more commonly occurs in women than in men [4]. HFI affects females 9 times more than it affects males and is mostly present among the middle-aged and elderly population [5]. The underlying mechanisms are not well known; however, there have been case reports where HFI is accompanied by endocrinopathies and or neuropsychiatric abnormalities [4].

In addition, a thickening of the skull has been associated with Paget's disease. Paget's disease is characterized by the destruction of the bone followed by reparative changes [6]. This disease often causes a boney enlargement of the skull and commonly involves the frontal and basal regions [7]. The new bone is laid down in a haphazard fashion and begins at the outer table of the skull; later, the differentiation between the inner and outer portions of the skull is lost and becomes thickened [7]. Paget's disease is often diagnosed in up to 8% of those over the age of 60 and affects both sexes; however, there is a slight male predominance [8]. Paget's disease patients with skull involvements have a 50% chance of developing a hearing loss.

Lastly, an infection that is often characterized by brain dysfunction is sepsis, which is associated with inflammation that may induce significant alterations to areas of the brain [9]. Specific areas that have been susceptible to ischemia in septic shock include the frontal junctional cortex, the dentate nucleus, hypothalamic nuclei, the amygdala, and the medullary autonomic nuclei [9]. Thus, in cadavers that have died of sepsis, one could expect abnormalities to occur in these respective areas of the skull.

The goal of this study is to observe the frequency of skull abnormalities in the cadavers that we have in the Gross Anatomy Laboratory. Furthermore, is to correlate these abnormalities to age and sex of the specimens.

## 2. Materials and Methods

The Gross Anatomy Laboratory at the University of Detroit Mercy, School of Dentistry collected data from 65 cadavers through 2016–2019, which were formalin embalmed. Out of the 65 cadavers, there were 34 females and 31 males. Reciprocating saws and mallets were used to separate the calvaria, approximating a sagittal plane above the superior orbital ridge in the anterior and external occipital protuberance posteriorly. All dura was removed on most specimens to get a better visualization of the inner surface of the frontal bone. Age and sex of each specimen containing the characteristic thickening of the frontal bone were recorded. Chief medical issues and cause of death of each cadaver were collected to determine presence of accompanying possible neuropsychiatric changes to the brain.

## 3. Results

**Figure 1:**
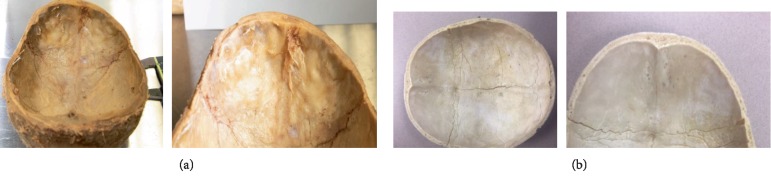
(a) Internal surface of the cadaver's frontal bone in a female skull, 68 years old. Note the bilateral thickening of the bone along both sides of the midline. (b) Internal surface of skull specimen's frontal bone with normal anatomical features.

Various skull disparities were observed among measurements in the 65 specimens collected. Out of the sixty-five cadavers obtained, nineteen specimens (29%) contained irregular, undulating, thickening on the internal surface of the frontal bone as shown in Figure 1. This finding closely resembled the gross anatomic appearance of HFI. Twelve of the nineteen specimens (63%) exhibiting frontal bone skull abnormalities were female ranging from 68 to 95 years old (35% of female cadaver sample). Seven of the nineteen specimens (37%) that showed a thickened skull with possible HFI were males ranging from 70 to 103 years old (23% of male cadaver sample).

The percentage of specimens exhibiting bone abnormalities in our study was much higher than the overall HFI prevalence observed in previous studies [7, 10] (Table 1). The average age of cadavers showing the skull abnormalities was 84.53 ± 9.29, which is similar to the average age of the overall female and male cadaver samples. The average age seems to be similar between males (82.14 ± 6.00) and females (85.92 ± 8.70). It is important to note that a great deal of variation was found between specimens.

In addition, five of the nineteen samples (26%) that presented with suspicions of HFI and a thickened skull had some form of neuropsychiatric abnormalities listed as the cadavers chief medical concerns, including Alzheimer's, dementia, depression, and Parkinson's disease; four out of these five specimens belonged to female cadavers. However, in the remaining sample, there were 10 cadavers (Table 1) diagnosed with neuropsychiatric abnormalities that had normal skull appearance. Thus, further monitoring for skull abnormalities in the Detroit Mercy Dental cadaver lab is planned.

## 4. Discussion

The current study examined various cadaver skulls over a 3-year data collection sample and noted abnormal thickening in the inner surface of the skulls, particularly the frontal bone. A common reason for said occurrence is hypertosis frontalis interna (HFI). There are other possible causes for thickening to occur in the frontal bone, such as Paget's disease. In previously conducted studies, the reported rates of HFI are higher in females (20%) than in males (3%) [7]. Another study [10] reported a prevalence of HFI as high as 13% in females compared to 1.3% in males. Thus, one can conclude that HFI is uncommon among males, and a significant difference exists between males and females. Even though both sexes had skull abnormalities present in our current study, the extent to which it occurred varied. Females tended to have more irregularities and thickening in the frontalis compared to males. Thus, it may be plausible to conclude from these differences that HFI is the cause not linked to one specific sex, but its severity is highly correlated to the female gender, with minor cases mostly occurring in males. The etiology of HFI is not well known, but it is suggestive that a hormonal imbalance is a likely cause. Studies have inferred that androgens do not particularly cause HFI to occur, rather the link to androgens and HFI is more related to the estrogen/androgen ratio imbalance, with a surplus of estrogen [11]. This may be a possible link as to why HFI is more prevalent in the female population, since females have an abundance of estrogen premenopause. Such effects that may take place early on in life may cause the thickening of the skull, which remains throughout the entirety of their lives, including postmenopausal females.

The presence of HFI may suggest a decrease in brain volume, which may indicate degenerative processes of the brain [11]. Furthermore, many of the female specimens with an increased severity of HFI had some form of neuropsychiatric abnormalities, including dementia, depression, Parkinson's, and Alzheimer's. As mentioned previously, Alzheimer's is linked to bone density. The extent and severity to which a patient has Alzheimer's can predispose them to having low bone mineral density [2]. This may suggest that the bodies' response to loss of brain matter and increased volume in the skull, is to increase bone deposition to minimize the movements of the brain and stabilize it within the capsule.

Some limitations of the study include a small sample size and a slightly higher number of female cadavers than males, which may decrease the statistical power of the study. Thus, the small sample size does limit the conclusions that may be drawn from this study. Another limitation is that the results are obtained from cadavers; thus, conclusions are limited to hypothesizing possible causes linking sex and HFI, since live studies cannot be performed on the specimens. Lastly, a radiological examination and pathology biopsy of the specimens were not available in the study limiting the conclusions to only suggestive of HFI.

Future studies must be done in order to gather more data to formulate conclusive results that are statistically significant and can be applied to the general population. With that said, to enhance the results obtained in this study, further research may be done to look at the relationship not only of sex and HFI, but HFI and the causes of death of specimens, such as prostate cancer prevalence and HFI and/or dementia prevalence and HFI. Overall, additional research needs to be performed in order to determine the other mechanisms that play a role in the structural changes of the skull that were seen in this study.

## 5. Conclusions

This study demonstrated the existence of a considerable number of anatomical abnormalities in the skull of cadavers of the Gross Anatomy Laboratory. These variations may be attributed to hypertosis frontalis interna, a condition that affects the skull of many in the aging population. The majority of skull abnormalities belonged to females. Investigation is needed for a definitive diagnosis of the found abnormalities. More cadaver studies will help to bolster sex differences and prevalence data and provide standardized measurements of lesions.

## Figures and Tables

**Table 1:** Prevalence, sex, and age of affected cadavers exhibiting frontal bone skull abnormalities and exhibiting neuropsychiatric disorders in our study.

	Male	Female	Total
Percentage of skull anomaly in the cadaver group	22.6%	35.2%	29%
	(7 out of 31)	(12 out of 34)	(19 out of 65)

Average age of cadavers	79.7 ± 12.6	85.2 ± 10.6	82.6 ± 11.8
	*n* = 31	*n* = 34	*n* = 65

Average age of affected cadavers	82.1 ± 6.0	85.9 ± 8.7	84.5 ± 9.3
	*n* = 7	*n* = 12	*n* = 19

Sex of affected skulls	36.8%	63.2%	100%
	*n* = 7	*n* = 12	*n* = 19

Overall prevalence of neuropsychiatric abnormalities	22.5%	23.5%	23.1%
	(7 out of 31)	(8 out of 34)	(15 out of 65)

Prevalence of neuropsychiatric abnormalities in affected cadavers	14.2%	33.3%	26%
	(1 out of 7)	(4 out of 12)	(5 out of 19)

Prevalence of neuropsychiatric abnormalities in remaining cadavers	25%	18%	22%
	(6 out of 24)	(4 out of 22)	(10 out of 46)

## Data Availability

The Data is available upon request.
